# Resistance to white spot syndrome virus in the European shore crab is associated with suppressed virion trafficking and heightened immune responses

**DOI:** 10.3389/fimmu.2022.1057421

**Published:** 2022-12-27

**Authors:** Rebecca S. Millard, Lisa K. Bickley, Kelly S. Bateman, Bas Verbruggen, Audrey Farbos, Anke Lange, Karen A. Moore, Grant D. Stentiford, Charles R. Tyler, Ronny van Aerle, Eduarda M. Santos

**Affiliations:** ^1^ International Centre of Excellence for Aquatic Animal Health, Cefas Laboratory, Weymouth, United Kingdom; ^2^ Sustainable Aquaculture Futures, University of Exeter, Exeter, United Kingdom; ^3^ Biosciences, Faculty of Health and Life Sciences, University of Exeter, Exeter, United Kingdom; ^4^ University of Exeter Sequencing Facility, Biosciences, Faculty of Health and Life Sciences, University of Exeter, Exeter, United Kingdom

**Keywords:** white spot disease (WSD), endocytosis, apoptosis, differential susceptibility, miRNAs, RNA-Seq, whiteleg shrimp, transcriptional response

## Abstract

**Introduction:**

All decapod crustaceans are considered potentially susceptible to White Spot Syndrome Virus (WSSV) infection, but the degree of White Spot Disease (WSD) susceptibility varies widely between species. The European shore crab *Carcinus maenas* can be infected with the virus for long periods of time without signs of disease. Given the high mortality rate of susceptible species, the differential susceptibility of these resistant hosts offers an opportunity to investigate mechanisms of disease resistance.

**Methods:**

Here, the temporal transcriptional responses (mRNA and miRNA) of *C. maenas* following WSSV injection were analysed and compared to a previously published dataset for the highly WSSV susceptible *Penaeus vannamei* to identify key genes, processes and pathways contributing to increased WSD resistance.

**Results:**

We show that, in contrast to *P. vannamei*, the transcriptional response during the first 2 days following WSSV injection in *C. maenas* is limited. During the later time points (7 days onwards), two groups of crabs were identified, a recalcitrant group where no replication of the virus occurred, and a group where significant viral replication occurred, with the transcriptional profiles of the latter group resembling those of WSSV-susceptible species. We identify key differences in the molecular responses of these groups to WSSV injection.

**Discussion:**

We propose that increased WSD resistance in *C. maenas* may result from impaired WSSV endocytosis due to the inhibition of internal vesicle budding by dynamin-1, and a delay in movement to the nucleus caused by the downregulation of cytoskeletal transcripts required for WSSV cytoskeleton docking, during early stages of the infection. This response allows resistant hosts greater time to fine-tune immune responses associated with miRNA expression, apoptosis and the melanisation cascade to defend against, and clear, invading WSSV. These findings suggest that the initial stages of infection are key to resistance to WSSV in the crab and highlight possible pathways that could be targeted in farmed crustacean to enhance resistance to WSD.

## 1 Introduction

White spot disease (WSD) outbreaks are a major limitation to production in penaeid shrimp aquaculture causing huge production and economic losses globally. White spot syndrome virus (WSSV), the pathogen causing WSD, can infect a broad range of species, including decapod crustaceans inhabiting waters outside tropical and shrimp farming regions (1–4). Whilst highly virulent in penaeid shrimp, other decapod hosts show widely different susceptibilities. Despite increasing efforts to understand WSD, the molecular and physiological traits that confer resistance and contribute to differences in WSSV susceptibility are not well established (5). Furthermore, the limited survival of many crustaceans with WSD presents difficulties identifying and grouping resistant individuals for comparative studies within these species. Comparisons between resistant and susceptible species, therefore, provide an alternative route towards understanding the molecular mechanisms of WSD resistance.

To date, studies comparing transcriptomic responses between WSSV-challenged resistant and susceptible species is limited to a comparison of *P. vannamei* and the freshwater prawn *Macrobrachium rosenbergii* (6). In that study, Peruzza et al. found many differentially expressed genes belonging to the immune system that were mostly up-regulated in *M. rosenbergii* and down-regulated in *P. vannamei* following WSSV infection, indicating differential immune responses between these species. They also found, exclusively in *P. vannamei*, several differentially expressed genes relating to moulting, indicating potential inhibition of the moult cycle either by WSSV or WSSV exposure in this species. These results offer a valuable contribution towards understanding the molecular basis of WSSV resistance. Developing our understanding of the molecular responses of resistant species to WSSV infection provides an important avenue for highlighting pathways and processes associated with WSD pathogenesis over the course of the infection, particularly in species that show limited WSD-related mortalities and pathology. Incorporating micro-RNA (miRNA) expression profiles, which are hypothesised to play a key role in invertebrate innate immune responses and widely recognised as important factors mediating the progression of diseases (7), into such studies is likely to add further to this understanding.

Experimental infections of *Carcinus maenas* (European shore crab) with WSSV have shown they have low susceptibility to WSD with limited disease pathology and mortality (4). Following infection, morphologically distinct virions are produced within *C. maenas*, but these still retain viability upon subsequent passage into a susceptible penaeid host. Minimal pathology is seen in *C. maenas* infected with WSSV with few nuclei displaying characteristic inclusion bodies. Furthermore, those that were present stain mostly with eosin, similar to that observed during early infection of susceptible hosts (4). These results are indicative of a slower progression of WSD in *C. maenas*, suggesting inherent resistance to WSSV and offer an opportunity to study how this resistance is mediated.

Here, we hypothesised that WSSV infection in *C. maenas* would result in temporal molecular responses, at the mRNA and miRNA levels, distinct from those observed in susceptible hosts, and that these differences have the potential to reveal some key molecular features responsible for WSD resistance in crustacea. To address these hypotheses, we identify the molecular responses of the WSSV recalcitrant host *C. maenas* by analysing the temporal changes in the mRNA and miRNA profiles following WSSV injection. Transcriptional changes, enriched KEGG pathways and gene ontology (GO) terms following WSSV challenge were identified and interactions of these transcripts with differentially expressed regulatory miRNAs were predicted. Comparison of the molecular responses of *C. maenas* (as a resistant species) to the molecular mechanisms underpinning disease progression in the highly susceptible *P. vannamei* was undertaken using a previously published dataset (8), in which global mRNA and miRNA transcription was investigated over the initial 36 hours following experimental WSSV infection. Our data demonstrate that the molecular responses of *C. maenas* to WSSV are distinct to those identified in *P. vannamei* and offer insights into the temporal differences that can be found between resistant and susceptible hosts infected with WSSV. The identification of key events leading to resistance to WSD provides a valuable resource for understanding and developing potential target pathways for disease treatment.

## 2 Materials and methods

### 2.1 Animal collection and husbandry

One hundred and twenty European shore crabs (*C. maenas*), that appeared healthy from their external appearance, were collected from the shoreline at Newton’s Cove, Weymouth, UK (50°34’ N, 02°22’ W) where WSSV is considered exotic and has never been detected. Crabs were kept in filtered, UV treated local seawater maintained at a constant salinity of 35 ppt, and temperature of 20 °C with a flow rate of 3-4 L/min. Crabs were housed in trough tanks at a density of 15 crabs per tank with physical separation to prevent individuals interacting with one another to avoid conflict. All animal procedures were carried out according to UK Home Office guidelines.

### 2.2 WSSV injection trial

A virus inoculum (WSSV UAZ 00-173B) was prepared as previously described by passaging through susceptible *P. vannamei*, which resulted in high replication of the virus (9) and sterile saline was prepared for control treatments. The final WSSV inoculum contained approximately 2.21 x 10^6^ virions/mg (quantified by qPCR).

**Figure 1:**
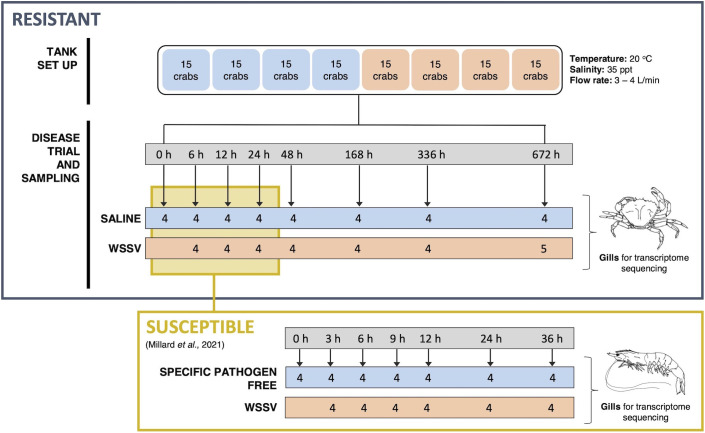
Schematic of the WSSV exposure and sampling procedure for both resistant and susceptible crustaceans. The upper panel outlines the experimental WSSV injection trial performed to generate RNA-seq and miRNA-seq datasets for resistant crab (*C. maenas*). Crabs were randomly allocated into two groups and housed in individual compartments in tanks. These groups were injected with either saline solution or WSSV-infected shrimp homogenate for the disease trial and the gills of 8 crabs were sampled from saline and WSSV-injected treatment groups at 0, 6, 12, 24, 48, 168, 336 and 672 hours and snap frozen for transcriptome sequencing. The lower panel illustrates the equivalent time course experiment in the susceptible shrimp (*P. vannamei*) reported in a previously published WSSV-exposure dataset utilised for comparison (8).

Crabs were divided into two treatment groups and injected at the base of the second walking leg with either WSSV inoculum or saline at a rate of 10 μl g^-1^ (wet body weight) equating to an average of 2.19 x 10^7^ virions per gram. Gill tissue was sampled from a minimum of 4 crabs per treatment group for transcriptome profiling and snap frozen in liquid nitrogen for storage at -80°C. Samples were taken throughout the 28-day (672 hours) exposure period at 0, 6, 12, 24, 48, 168, 336 and 672 hours post injection (hpi) (**Figure 1**). The selected time points reflect the extended period of time required for infection in resistant *C. maenas* compared to susceptible *P. vannamei*.

### 2.3 RNA extraction, library preparation and sequencing

RNA was extracted from gill tissues (n = 61, n = 4 or 5 per treatment time point and n = 4 for 0-hour time point) using the miRNeasy mini kit (Qiagen) with an on-column DNase I digestion (Qiagen). RNA integrity was assessed using the RNA 6000 Nano kit on the Agilent 2100 Bioanalyzer (Agilent). For mRNA sequencing (RNA-seq) RNA was spiked with External RNA Controls Consortium (ERCC) spike-in control mixes (Ambion) and mRNAs were purified using the NEB Poly(A)mRNA magnetic isolation module (NEB). Libraries were constructed using the Epicentre ScriptSeq v2 RNA-seq library preparation kit (Illumina) with Epicentre ScriptSeq Index PCR primers and the Agencourt AMPure XP system (Beckman Coulter) to purify cDNA. Libraries were pooled and sequenced to 100 bp in paired end mode on the Illumina HiSeq 2500. miRNA sequencing libraries (miRNA-seq) were produced using the gel-based Tru-Seq small RNA sample preparation kit (Illumina) with indexed adapters 1 – 36. Libraries were combined into two pools and sequenced across two lanes to 50 bp in single end mode on the Illumina HiSeq 2500.

### 2.4 Transcriptome analysis

To enable comparisons between datasets for resistant *C. maenas* and susceptible *P. vannamei* analyses of *C. maenas* mRNA and miRNA sequencing datasets were conducted as closely as possible to that described previously for *P. vannamei* (8). A detailed account of the transcriptome analysis is detailed in **Supplementary Figure 1**. Briefly, mRNA sequencing reads were demultiplexed and trimmed to remove adapters and low-quality bases using Trimmomatic v0.36 (10). WSSV transcription was assessed over time by determining the Bowtie2 v2.2.9 (RRID : SCR_016368) (11) alignment rates of trimmed sequencing reads to the WSSV-CN genome sequence [GenBank Accession: AF332093.3 (12)]. Crabs with a read alignment rate greater than 0.1% were considered to be infected with, and replicating, WSSV.

A transcriptome was assembled using the *de novo* assembler Trinity v2.4.0 specifying a minimum kmer coverage of 10 (RRID : SCR_013048) (13) and CD-HIT-EST v4.6 (using default parameters) (RRID : SCR_007105) (14) was employed to reduce transcript redundancy. Transcripts were annotated using Diamond v0.8.27 (RRID : SCR_016071) (15) and the blastx algorithm with e-value cut off of 1e^-5^ against the RefSeq release 85 complete protein database (RRID : SCR_003496). The final transcriptome was taxonomically filtered using MEGAN Community Edition (16) to retain viral and metazoan transcripts assumed to have arisen from the virus and host, respectively. Transcript abundance was estimated by read alignment to the transcriptome with Bowtie2 v2.2.9 (11) and RSEM v1.3.0 (RRID : SCR_013027) (17). Transcript expression was compared between control and WSSV treatment groups at each time point sampled using edgeR v3.16.3 (default settings) (RRID : SCR_012802) (18) with a Benjamini-Hochberg (19) corrected p-value threshold of < 0.05 for significant differential expression. Differential transcript expression was also compared by grouping time points where crabs displayed evidence of WSSV replication and assessing differences between animals replicating WSSV (WSSV-replicating) and those not replicating WSSV (WSSV-recalcitrant), and their grouped time point controls. Differentially expressed transcripts were annotated with Gene Ontology (GO) terms in Blast2Go PRO (RRID : SCR_005828) (20) and Gene Set Enrichment Analysis (GSEA) (RRID : SCR_003199) (21) was performed on lists of genes ranked based on log2 fold change to detect significantly enriched GO terms (with a minimum gene set size of 2 and adjusted p < 0.05). For KEGG pathway analysis, since there were no curated KEGG pathways for *C. maenas* available, *Drosophila melanogaster* pathways were used. *D. melanogaster* homologs were retrieved for all transcripts by Diamond blastx similarity to the Uniprot release 86.1 (RRID : SCR_018666) *D. melanogaster* complete proteome (evalue < 1e^-20^). Kyoto Encyclopaedia of Genes and Genomes (KEGG) (RRID : SCR_001120) bi-directional best hit annotations were retrieved for these homologs using the KEGG Automatic Annotation Server (KAAS) (22). The Generally Applicable Gene-set/Pathway Analysis (GAGE) package v3.9 (RRID : SCR_017067) (23) in R v3.6 (RRID : SCR_001905) (24) was applied with a significance cut off of 0.05 to determine which KEGG pathways were up- and down-regulated at each time point following WSSV injection. All plots were generated in R (24) using ggplot2 (RRID : SCR_014601) (25).

### 2.5 miRNA analysis

A detailed description of the tools and parameters applied to analyse the small RNA sequence dataset is provided in **Supplementary Figure 2**. Sequences were quality trimmed to remove adapters, low-quality bases and retain sequences of expected miRNA length (18 – 26 nt) using Cutadapt v2.1 (26). Reads were additionally filtered to remove sequences aligning to the *C. maenas* ribosomal mRNA sequence [GenBank Accession: EF035109.1 (27)] using Bowtie v1.0.0 (28), Samtools v1.4 (29), and Seqtk v1.2-r94 (30). Sequences with low complexity (*e.g.* homopolymers) were removed using Fqtrim v0.9.7 (31). miRNAs were predicted using the miRDeep2 pipeline v2.0.0.8 (RRID : SCR_010829) (32). To aid miRNA predictions, reads were aligned to a draft *C. maenas* genome sequence (33), the WSSV-CN genome and to known WSSV miRNAs downloaded from the VIRmiRNAv1.0. The miRDeep2 core algorithm was trained using 211 *Tribolium castaneum* mature miRNAs from the miRGeneDB v2.0 database (34) and 63 previously described *Penaeus japonicus* miRNAs (35). miRNAs with Rfam (RRID : SCR_007891) hits were discarded and the redundancy of the remaining sequences reduced with CD-HIT-EST (14). Predicted miRNAs were annotated using BlastN-short (36) against a customised database containing validated *T. castaneum*, *Drosophila melanogaster* and *Daphnia pulex* miRNAs from the miRGeneDB database (34) and previously described miRNAs from *P. vannamei* (37) and *Penaues japonicus* (35) with an e-value cut off of 1e^-5^. miRNAs with no hits were considered novel to *C. maenas* and annotated according to the following nomenclature Cma-pmiR-X where “pmiR” depicts a putative miRNA and “X” is a unique numeric identifier assigned to each miRNA. miRNAs were quantified using miRDeep2 (32) and differentially expressed miRNAs [with a Benjamini-Hochberg adjusted p-value < 0.05 (19)] were identified using DESeq2 v3.9 (RRID : SCR_015687) (38) to compare expression between WSSV-injected crabs and their time-matched controls. miRNA secondary structures were predicted using RNAfold v2.4.11 (RRID : SCR_008550).

### 2.6 Integrated analysis of RNA-seq and miRNA-seq

The ability of differentially expressed miRNAs to bind to mRNAs within the *C. maenas* transcriptome was determined by the overlapping predictions output by miRanda v3.3a (RRID : SCR_017496) (39) and RNA22 v2 (RRID : SCR_016507) (40). Reported predictions had minimum free energy scores < -20 kcal/mol and alignment within the seed region. The function of each miRNA was predicted by identifying significantly enriched GO terms within their list of predicted target transcripts using a Fisher’s Exact test with adjusted p-value cut off of 0.05.

### 2.7 Comparison of *C. maenas* and *P. vannamei* responses

A comparison of the transcriptional responses of *C. maenas* and *P. vannamei* to WSSV injection was performed by plotting the number and pattern of differentially expressed mRNAs and miRNAs at each time point post viral challenge. Overlapping miRNAs were identified by BLAST search of all differentially expressed *C. maenas* miRNAs against all differentially expressed *P. vannamei* miRNAs (evalue < 1e^-20^). Functional differences were inferred by visualising overlapping significantly enriched GO terms and up- and down-regulated KEGG pathways for each species using DiVenn (41).

## 3 Results

### 3.1 Disease presentation in resistant species

**Figure 2:**
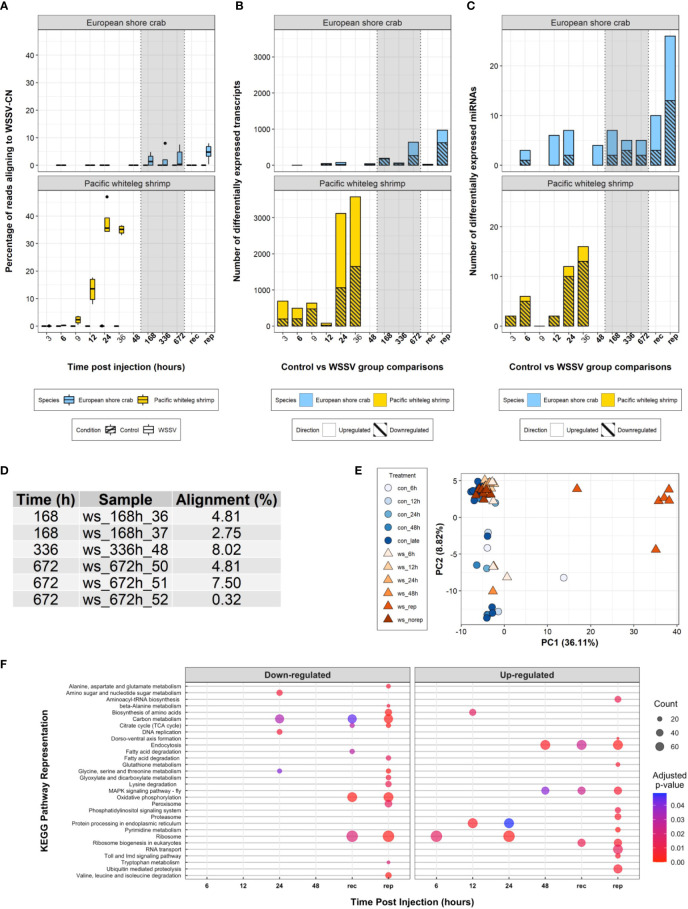
Transcriptome analysis. **(A)** Boxplot of the percentage of processed sequencing reads aligning to the WSSV-CN genome [GenBank Accession AF332093.3 (12)] over time in control and WSSV treatments. **(B)** A stacked bar plot of differentially expressed transcripts over time following WSSV injection in both *C. maenas* and *P. vannamei*. **(C)** A stacked bar plot of differentially expressed miRNAs over time following injection in both *C. maenas* and *P. vannamei*. **(D)** Percentage of processed sequencing reads aligning to the WSSV-CN genome in crabs displaying evidence for WSSV replication. **(E)** Principal component analysis of differentially expressed *C. maenas* transcripts in response to WSSV injection demonstrating separation of crabs displaying evidence for WSSV replication according to principal component 1. **(F)** Bubble plot of KEGG pathways up- and down-regulated over time in *C. maenas* in response to WSSV injection where point size represented the number of transcripts identified in the KEGG pathway and colour represents the adjusted p-value for enrichment.

During the 28-day experiment, no mortalities were recorded in either WSSV- or saline-injected crabs, and WSSV-injected crabs exhibited no obvious disease symptoms. Alignment of the trimmed mRNA sequencing reads to the WSSV genome suggests six of the WSSV-injected crabs from the 168 h (1 week) time point and onwards (consisting of two crabs at 168 h, one at 336 h and three at 672 h) (**Figures 2A, D**) were infected with, and replicating, WSSV at the time of sampling (the infection status of these individuals over time was unknown given that only terminal sampling was performed). The average proportion of sequences that were WSSV-derived in these six crabs was 4.7% (± 2.9, one standard deviation). In contrast, viral transcripts in highly susceptible *P. vannamei* were first observed at 3 h and increased from 0.3% to approximately 40% of expressed transcripts in the first 24h following infection (8).

### 3.2 Transcriptome assembly

A total of 648,542,863 reads were generated from 48 crab gill samples with an average of 9,443,887 reads pairs per sample (± 2,082,315, one standard deviation). A transcriptome was assembled using 538,301,558 100 bp trimmed reads to generate 184,222 reduced-redundancy transcripts (147,239 loci). The transcriptome had an N50 of 721 bp, Ex90N50 of 1,469 bp and average transcript length of 557 bp. On average, 94.4% of the sequencing reads from each sample were represented within the assembly. In addition, the assembly was largely complete, containing 89.0% of all BUSCO *Arthropoda* transcripts [single copy: 76.6%, duplicated:12.4%, fragmented: 8.5%, missing: 2.5%]. A total of 32,530 (17.7%) transcripts were annotated against the RefSeq complete protein database. Of these, 26,326 transcripts were extracted for downstream analyses which corresponded to 26,071 Metazoan and 255 Viral transcripts. The taxonomic groups with the greatest representation within these clades were *Pancrustacea* (13,636) and WSSV (228). Fifty seven percent of the filtered transcripts (15,070) were assigned GO annotations.

### 3.3 Differential transcription following WSSV injection

Eight hundred and twenty-nine differentially expressed transcripts were identified following WSSV injection in *C. maenas*. During the first 48 h few (< 80) transcripts were differentially expressed and during late infection (168 - 672 hpi) increased transcriptional differences were observed (**Figure 2B**). To elucidate the transcriptional responses of crabs displaying evidence of WSSV replication during late infection, crabs from 168 – 672 hpi were separated into groups, those that replicated WSSV (WSSV-replicating, n = 6) and those that did not (WSSV-recalcitrant, n = 7), and compared to saline-injected controls at these time points (n = 12). In this grouping, a total of 1,141 transcripts were differentially expressed over the exposure period with 993 transcripts differentially expressed during late infection; 25 were differentially expressed in WSSV-recalcitrant crabs and 968 in WSSV-replicating crabs (**Figure 2B**). A complete list of differentially expressed transcripts per time point is presented in **Supplementary Data Files 1-6**.

The pattern of differential transcript expression in *C. maenas* following WSSV injection differed substantially from *P. vannamei*. The biphasic response consisting of moderate alterations in transcription during early infection (from 3 – 12 hpi) followed by substantial transcriptional responses at the onset of disease in *P. vannamei* was not evident in *C. maenas;* for which limited changes in transcription occurred prior to 168 hpi. This lack of transcriptional separation between time points is also reflected in the principal component analysis (PCA) of **Figure 2E**. Similarities in the extent of the transcriptional response (i.e., large transcriptional response as WSSV infection progresses) were only observed in WSSV-replicating crabs. In addition, a PCA was performed based on the level of expression of the 1,141 differentially expressed transcripts and showed clear separation of WSSV-replicating crabs from both control and early-stage WSSV injected crabs along the first principal component, which explained 36.11% of the variation (**Figure 2E**).

### 3.4 miRNA prediction and expression

Small RNA sequencing produced 155,394,008 50 bp single end reads. Following quality trimming, and removal of both low complexity and overrepresented ribosomal RNA reads, 76,948,907 reads were retained averaging 1,282,482 (± 68,408 standard error of the mean) reads per sample. The remaining reads were within the expected size range of miRNAs (18 – 26 nt) (**Supplementary Figure 3**). In total, 13,640,252 unique small RNA reads were identified and 1,708,329 aligned to the *C. maenas* draft genome scaffolds. A total of 245 reduced redundancy *C. maenas* miRNAs were predicted with no rRNA or tRNA Rfam flags. Two hundred and twenty-one of these were associated with a significant RNAfold p-value (< 0.05) providing evidence for their ability to fold into unbifurcated hairpins. Fifty of the predicted miRNAs were assigned annotations from previously described *Penaeus japonicus* (n = 34) (35), *P. vannamei* (n = 7) (37), *Daphnia pulex* and *Tribolium castaneum* (n = 9) miRNA sequences (34). However, the majority of the predicted miRNAs (n = 195) did not show significant homology with any of the subsets of miRNAs utilised from related species (**Supplementary Data File 7**). A total of 104,810 reads aligned to the WSSV-CN genome but only one putative miRNAs was identified, which had a low miRDeep2 score (2.3), true positive rate of 61 ± 49% and no loop sequences, and was therefore not included in subsequent analysis.

From 6 – 672 hpi, 23 unique host miRNAs were significantly differentially expressed in response to WSSV injection (direct comparison between WSSV-injected and time matched controls). The number of differentially expressed miRNAs at each time point remained low (< 7), peaking at 12, 24 and 168 hpi. This low number of differentially expressed miRNAs was also observed in the first four time points of WSSV challenge in *P. vannamei*. However, in contrast to *P. vannamei* where the majority of miRNAs were upregulated in response to WSSV challenge, in *C. maenas* the majority of differentially expressed miRNAs were downregulated.

Crabs were again grouped during late-stage infection (168 - 672 hpi), as for the RNA-seq analysis based on their ability to replicate WSSV (determined *via* presence of RNA reads aligning to the WSSV-CN genome), and here 36 unique differentially expressed miRNAs were detected. WSSV-recalcitrant crabs displayed a relatively low number of both upregulated and downregulated miRNAs compared to each of the early time point time-matched comparisons. Additionally, consistent with the mRNA-seq dataset, the number of differentially expressed miRNAs in WSSV-replicating crabs was substantially higher (n = 26) with an equal number of up- and downregulated miRNAs. Functional information represented herein refers to the 36 differentially expressed miRNAs resulting from WSSV replication-based groupings (**Supplementary Data File 8**).

There was minimal (n = 7) overlap in the differentially expressed miRNAs detected in shrimp and crabs in response to WSSV challenge. Three of these miRNAs were differentially regulated in both shrimp and crabs. This included miR- 279d which was upregulated in WSSV-replicating crabs, but downregulated at 24 hpi in shrimp, and miR-133 which was upregulated during early infection in shrimp (3 and 6 hpi) but downregulated at 48 hpi in crabs. Putative novel miRNA labelled Pva-pmiR-118 in shrimp and Cma-pmiR-14 in crabs was also differentially regulated, with significant increases in transcription in shrimp (6 hpi) and downregulation in crabs (WSSV-replicating) indicating that it may play an important role in WSSV defence.

### 3.5 Functional analysis of transcriptome and miRNA data

Analysis of GO term enrichment both for the direct time point comparisons from 6 – 48 hpi and from WSSV-replicating/recalcitrant groups during late infection revealed 91 enriched GO terms (**Supplementary Figure 4**). In addition, 30 unique KEGG pathways were significantly perturbed during the experiment (**Figure 2F**). A complete list of significantly enriched GO terms and perturbed KEGG pathways is presented per time point in **Supplementary Data Files 9 and 10**. The 36 differentially expressed miRNAs from WSSV replication-based comparisons were predicted to target a total of 2,349 unique mRNAs (**Supplementary Data File 11**). Some miRNAs were predicted to target the same mRNAs resulting in a total of 3,551 miRNA-mRNA interactions predicted by both RNA22 and miRanda. Among the lists of mRNAs targeted by each miRNA, 210 GO terms were enriched. In comparison to *P. vannamei*, the number of enriched GO terms was higher in *C. maenas* (n = 91 and n = 35, respectively) and the number of significantly represented KEGG pathways was lower (n = 30 versus n = 43, respectively).

### 3.6 Early response to WSSV injection

During early infection (6 – 48 hpi), a limited number of differentially expressed transcripts and miRNAs were detected (**Figures 2B, C**). At 6 hpi, two novel *C. maenas* miRNAs with hypothesised roles in the regulation of intracellular trafficking and apoptotic processes were significantly downregulated. The first, Cma-pmiR-32 was consistently significantly downregulated from 6 – 24 hpi (6 h: logFC = -3.17, FDR = 0.022; 12 h: logFC = -3.39, FDR = 0.007; 24 h: logFC = -3.30, FDR = 0.009). Among the transcripts predicted to bind to Cma-pmiR-32 was dynein light chain roadblock-type-2-like which connects the dynein 2 molecular motor to cargo vesicles for transport along microtubules (42). This transcript followed a trend for upregulation at both 6 and 12 hpi and its reduced posttranscriptional silencing may therefore aid WSSV entry and intracellular movement. The second, Cma-pmiR-172, was also consistently downregulated from 12 - 48 hpi (12 h: logFC = -3.77, FDR = 4.21x10^-6^; 24 h: logFC = -3.03, FDR = 0.001; 48 h logFC = -2.96 FDR = 0.002) and in WSSV-replicating (logFC = -3.95, FDR = 1.70x10^-13^) and WSSV-recalcitrant crabs (logFC = -2.88, FDR = 1.37x10^-7^). However, Cma-pmiR-172 was predicted to target 150 transcripts with significant enrichment for the GO term apoptotic process (FDR = 0.042). In addition, among the targeted transcripts, dynamin-like 120kDa protein mitochondrial, which participates in mitochondrial remodelling and the release of cytochrome c to induce apoptosis (43) may increase apoptotic rate in *C. maenas* to protect against WSSV infection.

Subsequently, at 12 hpi, 45 transcripts were differentially expressed, with no significantly enriched GO terms. Strikingly, among these transcripts, dynamin-1like protein isoform X3, a regulatory GTPase for the control of endocytosis *via* clathrin-mediated endocytosis (44), was strongly downregulated (logFC = -7.48, FDR = 3.17x10 ^-2^). As clathrin-mediated endocytosis is a dynamin-dependent entry route hijacked by WSSV (45), this downregulation has the potential to strongly impede WSSV entry in *C. maenas*. Concomitantly, at 12 hpi various transcripts previously associated with increased WSSV pathogenesis were upregulated including the key glycolytic enzyme triosephosphate isomerase (TPI) (logFC = 3.18, FDR = 2.05x10^-2^), WSSV-interacting peritrophin-48-like (logFC = 4.33, FDR = 3.74x10^-2^) and Kruppel homolog-1 (logFC = 3.91, FDR = 7.68x10^-7^). This suggests that while some molecular pathways repress infection, transcripts that promote infection were simultaneously altered. At 12 hpi, all differentially expressed miRNAs were downregulated and none displayed homology to previously described sequences. This included novel Cma-pmiR-167 (logFC = -3.28, FDR = 5.10x10^-3^), predicted to target 137 transcripts with functional roles in posttranscriptional gene silencing by RNA (FDR = 0.011), protein lipoylation (FDR = 0.004) and ecdysone binding (FDR = 0.011). As Cma-pmiR-167 expression was significantly decreased, the expression of targets, like putative helicase MOV-10 isoforms X1 and X3, increased. As MOV-10 knockdown has previously been linked to increased WSSV susceptibility (46) the decreased expression of this miRNA may contribute to increased WSSV recalcitrance in crabs.

By 24 hpi, the number of differentially expressed transcripts increased to 78, with significant enrichment in four GO terms including serine-type peptidase activity (FDR = 0.036) and proteolysis (FDR = 0.042). Six transcripts contributing to the enrichment of these terms were associated with the melanisation cascade, an important innate immune response of crustaceans associated with WSSV survival (47), and the downregulation of 5 of these indicates its potential suppression upon WSSV challenge. For example, the significant downregulation of melanisation protease 1-like isoform X2 (logFC = -10.28, FDR = 0.001), a serine protease that activates phenoloxidase (PO) to catalyse the production of melanin which encapsulates invading pathogens, may reduce WSSV virion clearance. Similarly, the downregulation of trypsin 1-like (logFC = -9.57, FDR = 3.07 x 10^-6^)) and trypsin I-P1-like (logFC = -7.51, FDR = 4.41 x 10^-6^) may limit PO activity increasing WSSV susceptibility (47). During this time point, 7 miRNAs were also differentially expressed, including significantly upregulated Cma-miR-92b (logFC = 1.24, FDR = 0.025). This miRNA was predicted to target WSSV transcript E3 ligase, immediate-early protein suggesting that it may directly impair the translation of viral transcripts. As the transcription of early and late WSSV genes depends on the translation of immediate early genes, this has the potential to significantly disrupt or delay the WSSV infection process in *C. maenas*. The significant upregulation of this miRNA in WSSV-recalcitrant crabs (logFC = 0.77, FDR = 0.042) further supports its hypothesised role in WSSV defence and contribution to WSD resistance.

At the final ‘early’ time point following WSSV injection in *C. maenas* (48 hpi), 44 transcripts with broad enrichment of GO terms such as purine ribonucleoside triphosphase binding (FDR = 0.002) and ion binding (FDR = 0.005), and 7 miRNAs were differentially expressed. Several cytoskeletal transcripts such as muscle LIM protein Mlp84B-like isoform X8 (logFC = -9.57, FDR = 3.47x10^-2^), actin, muscle-like isoform X1 (logFC = -10.40, FDR = 3.99x10^-2^) and zinc finger MYM-type protein 3 isoform X4 (logFC = -8.27, FDR = 4.67x10^-2^) were downregulated at 48 hpi - indicating that transport of substances, including invading WSSV, throughout the intracellular environment is likely to be disrupted within the crab. In addition, the downregulation of miRNA Cma-miR-133 (logFC = -5.40, FDR = 0.006), predicted to target transcripts PH-domain containing protein 6-like isoform X2, ral GTPase-activating protein beta-like and befeldin A-inhibited guanine nucleotide exchange protein 3 with functional roles in the regulation of small GTPase mediated signal transduction (FDR = 0.013) may affect multiple cellular signalling processes including cytoskeletal reorganisation and phagocytosis (48).

However, several transcripts with potential roles in enhancing WSSV infection were also significantly altered, such as increased cuticle protein AMP1A-like (logFC = 11.35, FDR = 1.23x10^-2^) which interacts with VP24 and whose knockdown in linked to decreased WSSV mortality (49); decreased immune-related heat shock protein 83-like (logFC = -8.38, FDR = 3.99x10^-2^); and increased RNA-directed DNA polymerase from mobile element jockey-like (logFC = 2.54, FDR = 3.04x10^-2^) which indicates an increase in both RNA transcription and DNA replication, possibly to enhance WSSV production. Collectively, these suggest the regulation of a broad array of processes at 48 hpi.

### 3.7 Late responses to WSSV injection

#### 3.7.1 WSSV-replicating crabs

The largest transcriptional response was observed in WSSV-replicating crabs, with 968 mRNAs and 26 miRNAs differentially expressed. The latter included 11 putative novel miRNAs that had not previously been described. An equal number of miRNAs in WSSV-replicating crabs were upregulated and downregulated, and 20 of these were exclusively differentially expressed in WSSV-replicating crabs.

WSSV-derived transcripts were well-represented in WSSV-replicating crabs, with 203 significantly differentially expressed, resulting in enrichment of the GO terms viral membrane (FDR < 0.001), viral envelope (FDR < 0.001), and virion (FDR < 0.001). The concomitant enrichment of GO terms deoxyribonucleotide metabolic process (FDR = 6.13x10^-4^) and deoxyribonucleotide biosynthetic process (FDR = 8.46x10^-4^) suggest active DNA replication and the presence of a proliferating virus like that observed from 6 hpi in *P. vannamei*. These included upregulated transcripts associated with DNA replication originating from both virus (*e.g.*, thymidylate synthetase (logCPM = 8.14) and host (*e.g.*, ribonucleoside-diphosphate reductase subunit M2-like isoform X1 (logFC = 5.35, FDR = 3.78x10^-10^)). The initiation of viral protein synthesis may also be enhanced by the decreased posttranscriptional silencing of mRNA eukaryotic translation initiation factor 3 subunit A-like which is involved in the initiation of protein synthesis (50), as its predicted posttranscriptional regulator Cma-miR-276 is downregulated (logFC = -0.95, FDR = 9.75x10^-4^).

Several functional differences could contribute to these crabs’ ability to replicate WSSV, including an enhanced ability for WSSV to enter cells conferred by the downregulated miRNA Cma-miR-275 (logFC = -0.85, FDR = 7.50x10^-4^). Forty-nine predicted Cma-miR-275 mRNA targets were enriched for GO terms vesicle uncoating (FDR = 4.00x10^-2^), clathrin coat disassembly (FDR = 4.00x10^-2^), and phosphatidylinositol-3,4,5-triosphate 3-phosphate activity (FDR = 4.00x10^-2^) and included cyclin-G-associated kinase. Reduced posttranscriptional silencing of this transcript, which functions to support the uncoating of clathrin coated vesicles (51), may therefore enhance WSSV entry which occurs *via* multiple endocytic routes. Following successful entry, the enrichment of GO term cytoskeleton (FDR = 2.27x10^-2^) attributed to by the upregulation of transcripts involved in intracellular movement such as tubulin beta chain-like isoform X1 (logFC = 2.20, FDR = 1.25x10^-2^) and myosin-I heavy chain isoform X2 (logFC = 1.35, FDR = 2.14x10^-2^) may enhance WSSV transport to the nucleus for replication. This direction of change in cytoskeletal transcripts contrasts the downregulation observed in *C. maenas* at 48 hpi but agrees with that observed in susceptible *P. vannamei* during early infection.

Similarly, the enrichment of ion transport (FDR = 2.15x10^-2^) and inorganic molecular entity transmembrane transporter activity (FDR = 3.46x10^-3^) GO terms which included downregulated V-type proton ATPase 16kDa proteolipid subunit (logFC = -1.38, FDR = 4.27x10^-2^) and V-type proton ATPas 116 kDa subunit a isoform X1 (logFC = -2.40, FDR = 2.90x10^-2^) may enhance WSSV transport. Downregulation of ATPase subunits were also reported for *P. vannamei* at 9 hpi and hypothesized to result in decreased acidification of vesicles which may enable WSSV to evade the lysosome and increase availability of ATP to aid viral replication. However, *C. maenas* may counteract this effect with the significant downregulation of Cma-pmiR-14 (logFC = -0.70, FDR = 9.04x10^-3^) resulting in the reduced posttranscriptional silencing of target V-type proton ATPase subunit S1-like, and other predicted targets contributing to the enrichment of GO term ATPase activity, coupled to transmembrane movement of ions, rotational mechanism (FDR = 2.45x10^-2^). The increased posttranscriptional silencing of vacuolar protein sorting-associated 54-like which contributes to the formation of golgi-associated retrograde transport complex (52) by upregulated Cma-miR-153 (logFC = 1.21, FDR = 1.91x10^-2^) may also disrupt endosome maturation and sorting.

WSSV-replicating crabs may also alter WSSV entry at the nucleus *via* contrasting regulation at the mRNA and miRNA level. Firstly, nuclear envelope pore complex transcripts such as Nup205-like (logFC = 1.15, FDR = 3.15x10^-2^), and Nup155-like (logFC = 1.75, FDR = 4.89x10^-2^) were significantly upregulated, with enrichment in GO terms nuclear envelope (FDR = 2.10x10^-2^) and nuclear pore (FDR = 2.10x10^-2^) suggesting increases in nuclear pore complexes that may facilitate nuclear penetration. In contrast, the increased posttranscriptional silencing of mRNA targets of Cma-pmiR-novel_20 (logFC = 1.72, FDR = 1.84x10^-7^) may counteract this effect. These targets include importin-4-like isoform X2, a nuclear transport receptor responsible for protein transport into the nucleus (53) which contributed to the significant enrichment of GO terms protein import into nucleus (FDR = 5.27x10^-3^), and protein localization to nucleus (FDR = 5.27x10^-3^). The increased posttranscriptional silencing of these transcripts may therefore lead to a reduction in nuclear transporters, reduced WSSV imports and increased WSSV resistance. Cma-pmiR-novel_20 is likely to alter a broad range of responses as it is predicted to target 463 mRNAs with enrichment in 20 GO terms. Of note, the GO term positive regulation of actin nucleation (FDR = 2.10x10^-2^) and contributing transcript neural Wiskott-Aldrich syndrome protein-like, a transcript that regulates actin polymerization (54) may also impede WSSV entry.

#### 3.7.2 WSSV-recalcitrant crabs

In contrast to WSSV-replicating crabs, the 7 WSSV-recalcitrant crabs exhibited similarly low levels of transcriptional change to early-stage time matched control comparisons with 25 mRNAs and 10 miRNAs differentially expressed.

The 25 differentially expressed transcripts were not enriched for any GO terms or KEGG pathways, however, they included several significantly upregulated mRNAs involved in immune function that may result in enhanced protection against WSSV infection. These included anti-WSSV protein hemocyanin-like (logFC = 6.62, FDR = 2.39x10^-2^) which binds to major coat protein VP28 and inhibits transcription of *wsv06* and *wsv421* (55), antiviral apoptosis activator caspase-1-like isoform X2 (logFC = 5.46, FDR = 2.06x10^-2^), and cell death-inducing p53-target protein 1 (logFC = 2.59, FDR = 4.54x10^-2^). In addition, the significant downregulation of Cma-miR-193a (logFC = -6.89, FDR = 3.89x10^-5^) may promote apoptosis and alter NF-κB and Wnt immune-related signaling cascades (56–58) to enhance WSSV clearance through reduced posttranscriptional silencing of its target transcripts. These included death-associated protein kinase-1like, ubiquitin protein ligase E3A putative, ubiquitin carboxyl-terminal hydrolase 10, and HMG domain-containing protein 4 isoform X1. This is in direct contrast to *P. vannamei* which demonstrated significant upregulation of Pva-miR-193 at the onset of WSD symptoms at 24 hpi.

In parallel to the hypothesized WSSV-reducing transcription changes, several transcriptional changes with potential WSSV infection-enhancing properties were of note in WSSV-recalcitrant crabs. For example, the downregulation of antioxidant superoxide dismutase [Cu-Zn]-like (logFC = -6.05, FDR = 4.36x10^-2^), putative glutathione-specific gamma-glutamylcyclotransferase 2 (logFC = -5.51, FDR = 4.42x10^-2^) which depletes glutathione to promote apoptosis in vertebrates (59), and zinc finger CCCH domain-containing protein 18 (logFC = -4.53, FDR = 2.30x10^-2^) which has probable antiviral activity (60). However, the similar pattern of expression of these transcripts in both WSSV-replicating and WSSV-recalcitrant crabs indicates that they are unlikely to explain the observed differences in replication between these groups and could mean WSSV-recalcitrant crabs have not fully cleared the invading virus. The downregulation of novel miRNA Cma-pmiR-32 (logFC = -1.94, FDR = 2.35x10^-2^), which was also significantly downregulated during early time points, may also play a role in enhancing WSSV entry as previously described.

Finally, one novel putative miRNA, Cma-pmiR-40 (logFC = -2.13, FDR = 4.98x10^-2^), was exclusively differentially expressed in WSSV-recalcitrant crabs and may therefore play a key role in WSSV resistance. This miRNA was predicted to target 20 mRNAs including DNA replication factor Cdt1-like, scaffold attachment factor B2 isoform X2, heparan sulfate 2-O-sulfotransferase 1-like, translation elongation factor 2-like, and RNA-directed DNA polymerase from mobile element jockey-like.

### 3.8 Comparison with *P. vannamei* GO enrichment and KEGG representation

**Figure 3:**
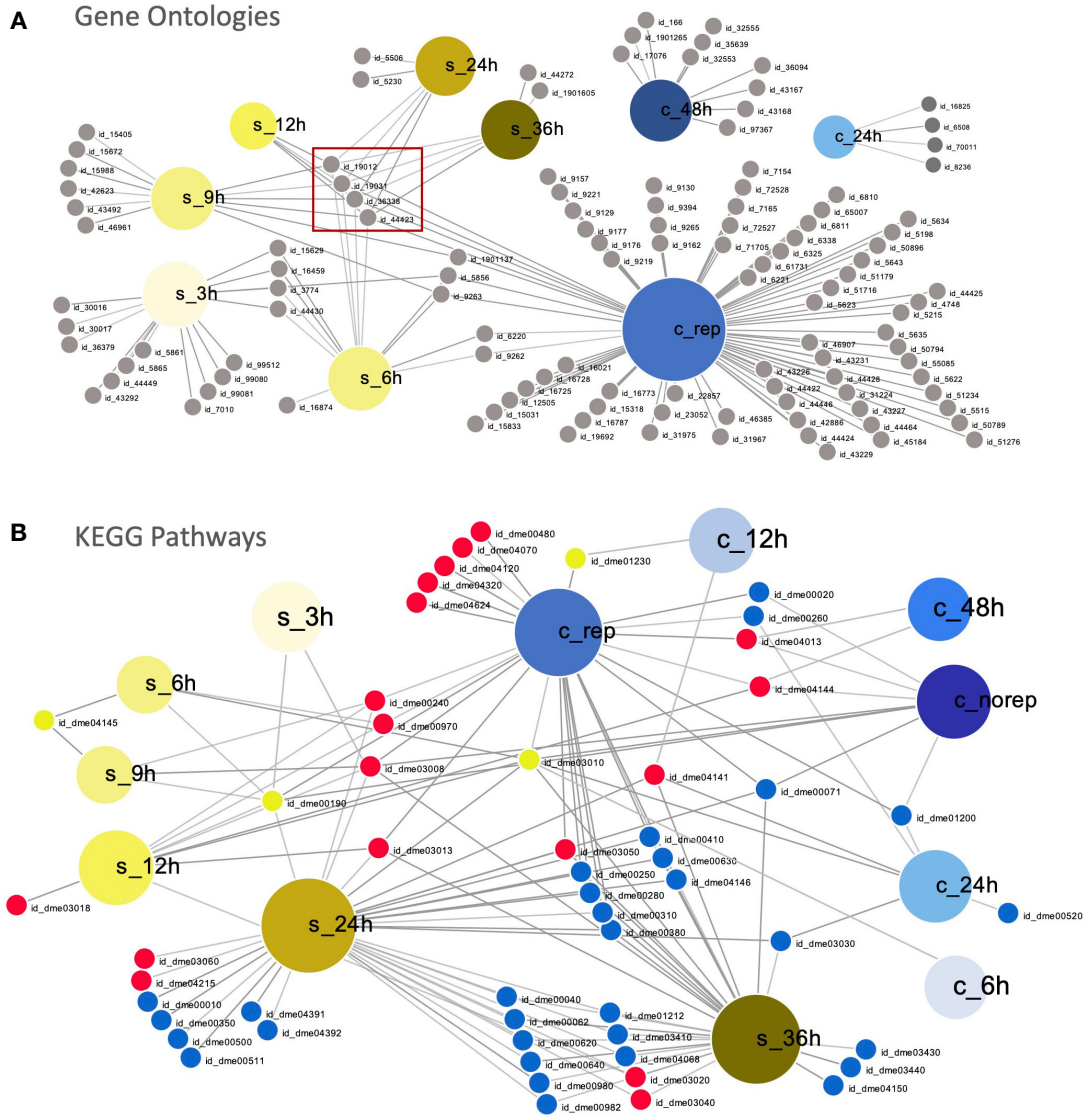
Comparison of susceptible and resistant host responses to WSSV infection. **(A)** Visual representation of overlapping significantly enriched GO IDs detected in WSSV-injected *C. maenas* (represented by large blue nodes with ‘c_’ labels) compared to susceptible *P. vannamei* (represented by large yellow nodes with ‘s_’ labels). Each GO ID is represented by a grey node connected to a large node representing the time point and species within which this term was significantly enriched. A table of the GO IDs associated with each time point is presented in **Supplementary File 10**. The central cluster of virus-related GO terms is indicated with a red box. **(B)** Overlapping KEGG pathways in WSSV-injected *P. vannamei* (represented by large yellow nodes with ‘s_’ labels) and *C. maenas* (represented by large blue nodes with ‘c_’ labels). Each KEGG ID is represented by a small node connected to a large node representing the time point and species within which this pathway was significantly enriched. Down-regulated KEGGs are depicted by small blue nodes, down-regulated KEGGs are represented by small red nodes and shared KEGGs that were both up- and down-regulated are depicted by yellow nodes. A table of the KEGG pathway IDs associated with each time point is presented in **Supplementary File 11**. These plots were created using DiVenn (41).

The overlapping enriched GO terms during early (6 – 48 hpi in *C. maenas* and 6 – 12 hpi in *P. vannamei*) and late (grouped by WSSV replication status in *C. maenas* and 24 – 36 hpi in *P. vannamei*) WSSV infection are displayed in **Figures 3A, B**, respectively. During early infection, the short time between sampling points in the *P. vannamei* experiment capture the dynamic progression of responses to infection which are linked by a central cluster of virus-related GO IDs which continues into the late time points (**Figure 3A**). In contrast, enriched GO terms for 24 and 48 hpi in *C. maenas* cluster separately from each other and from shrimp time points. During late infection the number of enriched GO terms in WSSV-replicating crabs is much larger than in shrimp whose enriched GO terms are somewhat limited despite the large number of differentially expressed transcripts associated with these. GO terms such as cytoskeleton (GO:0005856), deoxyribonucleotide metabolic process (GO:0009265) and deoxyribonucleotide biosynthetic process (GO:0009263) and carbohydrate derivative biosynthetic process (GO:1901137) displayed overlap between early time point shrimp (3 – 9 hpi) and WSSV-replicating crabs. In contrast, no significantly enriched GO terms were detected in late-stage WSSV-recalcitrant crabs.

At the level of enriched KEGG pathways, distinct responses were also observed in the early responses to WSSV injection in shrimp and crabs (**Figure 3B**). Overlap was observed between shrimp and crabs in the amino sugar and nucleotide sugar metabolism pathway (dme00520), which was up-regulated in shrimp and down-regulated in crabs. Further to this, the ribosome (dme03010) KEGG pathway was also differentially represented, with up-regulation during the initial 6 hpi in both species, and down-regulation at 12 and 24 hpi in shrimp and crabs, respectively. At late time points a greater degree of overlap was observed between the KEGG pathways represented within the datasets with the majority of shared KEGGs being either up- or down-regulated in both shrimp and crabs. However, several KEGG pathways differed in their representation including ribosome (dme03010) and oxidative phosphorylation (dme00190).

## 4 Discussion

**Figure 4:**
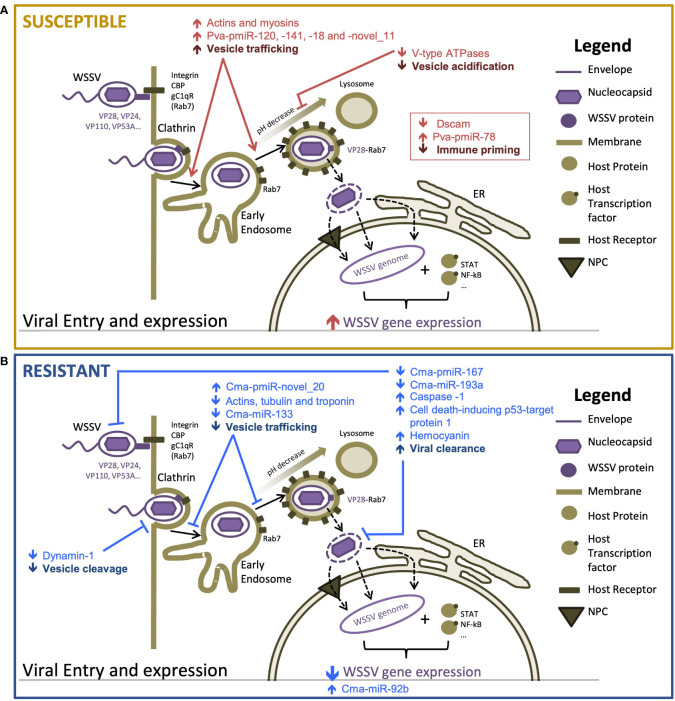
Proposed mechanisms responsible for differences in susceptibility to WSSV between resistant and susceptible hosts [adapted from Verbruggen et al. (61)] **(A)** highly susceptible *Penaeus vannamei* and **(B)** highly resistant *Carcinus maenas*. Host components are displayed in brown tones and viral components are displayed in purple tones. Arrows indicate the direction of change in transcription or activity of a proposed process. Differential responses of susceptible and resistant hosts are shown in red and blue, respectively.

We provide the first detailed description of the molecular (mRNA and miRNA) responses of WSD-recalcitrant *C. maenas* to a WSSV injection challenge. We used these datasets to identify similarities and differences in the temporal molecular responses compared with that in a highly WSD-susceptible and economically important species, *P. vannamei*. The key differences in the early responses to WSD in crustaceans with differential susceptibility are summarized in **Figure 4**. We identify probable mechanisms of increased WSD recalcitrance whereby WSSV entry and intracellular movement is firstly impeded during the initiation of the infection process and, subsequently, innate immune responses including apoptosis promoting and anti-WSSV miRNAs are bolstered to delay and counteract the WSSV infection process.

### 4.1 Early transcriptional responses to WSSV suggest that suppressed viral entry and intracellular movement are important mechanisms of resistance to WSD

Limited transcriptional responses exhibited during early infection in *C. maenas* indicated that crabs were not experiencing stress associated with disease progression as observed in *P. vannamei*. This was supported by the absence of both mortalities and WSD symptoms throughout the 28-day challenge, an observation consistent with previous studies (1, 4). The absence of WSSV transcripts from crabs in the first 48 hpi indicates that the infection process is hindered prior to nuclear invasion and WSSV replication, which typically occurs from 6 hpi in highly susceptible hosts (62). We, therefore, propose that the initial line of WSSV defense elicited by *C. maenas* operates by reducing WSSV entry. Typically, entry occurs *via* several endocytic routes, including clathrin-mediated endocytosis, caveolae-mediated endocytosis and micropinocytosis (45, 63, 64). The former is regulated by GTPase dynamin-1 (65), which is responsible for super-constriction of the plasma membrane to enable internal budding off of clathrin-coated vesicles (66). The strong downregulation of dynamin-1 at 12 hpi is therefore likely to impede viral entry and the infection process using the same mechanism as *Cherax quadricarinatus* which displayed reduced WSSV entry and transcription following dynamin enzymatic inhibition and RNAi knockdown (63).

Increased WSD recalcitrance may subsequently be supported by a reduction in the transcription of cytoskeleton components at 48 hpi. The cytoskeleton is reported to play a key role during the WSSV infection process (67). For example, microfilaments tubulin and actin have previously been shown to enhance WSSV entry and movement *via* binding to γ-aminobutyric acid receptor-associated protein (GABARAP) and multiple WSSV proteins such as VP28, which promotes WSSV interaction with the cytoskeleton (63). Here we show that actins, tubulins and troponin-like transcripts are downregulated during early infection suggesting that the transport of substances, including invading WSSV, throughout the intracellular environment is likely to be suppressed within crab cells. This downregulation directly contrasts the expression patterns reported in previous WSSV exposures from 1.5 and 3 hpi in susceptible *P. vannamei* (6, 8). In addition, the upregulation of cytoskeletal transcripts in WSSV-replicating crabs in this study further supports their requirement for facilitating infection. Subsequent to the reduced interaction of WSSV with the cytoskeleton proposed here, the formation of actin fibers, which provide an internal network for the virus to move along, may be impeded by increased negative regulation of neural Wiskott-Aldrich syndrome protein-like, a regulator of actin polymerization (54), by Cma-pmiR-novel_20. By delaying the entry and movement of virions towards the nucleus for replication, the host benefits from increased time to initiate further defense mechanisms to protect against infection. Together, these data document the multiple mechanisms potentially utilized by crab cells to delay viral entry and transport to the nucleus, serving as an effective first barrier to disease progression.

### 4.2 Multiple miRNAs likely confer increased WSSV resistance by fine-tuning apoptosis and halting early viral transcription

The second hypothesized line of WSSV defense in *C. maenas* may operate *via* regulating innate immune responses such as apoptosis and WSSV-defensive miRNAs. Apoptosis is a defense mechanism that must be finely tuned to ensure its defensive role detriments the virus and not the host. The action of several miRNAs during early infection (including Cma-pmiR-172, Cma-miR-92b and Cma-miR-193a) may regulate this process in *C. maenas*. The latter was predicted to target transcripts which function in the regulation of apoptosis and protein ubiquitination to attenuate signaling cascades such as NF-κB (68), death-associated protein kinase 1-like and ubiquitin protein ligase E3A putative. Therefore, their increased negative regulation by Cma-miR-193, during early infection (6 hpi) has the potential to profoundly disrupt the WSSV infection process by altering immune-related signaling cascades to protect against WSSV. This is supported by evidence that silencing of the predicted target, death-associated protein 1, results in reduced WSSV copy number and WSSV-induced apoptosis in *P. japonicus* hemocytes (69). In WSSV-exposed *P. japonicus* (35), *Procambarus clarkii* (70), and *P. vannamei* (8) miR-193 was also upregulated. In the latter, upregulation occurred at 24 hpi indicating that the hypothesized protective effects of this miRNA are effective only when elicited prior to WSSV replication. Finally, the reduced expression of Cma-miR-193a in WSSV-recalcitrant crabs may promote apoptosis of infected cells and the clearance of invading virions. Increased Cma-miR-92b at 24 hpi and in WSSV-recalcitrant crabs may provide further generic immune protection from WSSV as this miRNA has been shown to induce apoptosis in shrimp challenged with Acute Hepatopancreatic Necrosis Disease-causing *Vibrio parahaemolyticus* and non-lethal heat shock (71). However, perhaps most significant is the potential for Cma-miR-92b to target the viral transcript E3 ligase, immediate-early protein, suggesting that this miRNA may be able to directly impair the translation of viral transcripts during early infection. As the transcription and translation of early and late WSSV genes depends on the translation of immediate early genes, this has the potential to significantly disrupt or delay the WSSV infection process in *C. maenas*. The absence of WSSV transcripts in crabs at these time points, and contrasting downregulation of miR-92 in susceptible WSSV-injected *P. japonicus* (35) further supports this hypothesis. Finally, decreased post-transcriptional silencing of MOV-10 isoforms, a gene whose knockdown has previously been linked with WSSV susceptibility (46), by Cma-pmiR-167 may further contribute to increased WSD recalcitrance in crabs.

### 4.3 Initiation of melanization cascade may determine heterogeneity in WSSV replication among crabs

WSSV-injected crabs exhibited individual variability that may determine whether they replicate WSSV. A reduction in expression of transcripts associated with the melanization cascade, an important innate immune response of crustaceans (72), during early infection (24 hpi) may be a key determinant of whether WSSV-challenged crabs will go on to replicate WSSV. This is supported by observations that the melanization response is attenuated by WSSV during the infection process, to enhance WSSV infection in shrimp (47, 73). This process seems to take around 7 days (168 hpi) to establish if crabs are to replicate the virus whereby, an average of 4.7% transcripts within *C. maenas* were WSSV-derived. This indicates that once in the nucleus, the increase in WSSV-derived transcripts (and likely associated replication of the virus) occurs over a long period of time, suggesting that *C. maenas* can elicit further responses to limit WSSV transcription. In highly susceptible species, that do not possess effective mechanisms to prevent virus replication, such as *P. vannamei*, viral transcripts can account for up to ~40% of the total transcripts (8).

The transcriptional response of WSSV-recalcitrant crabs is underpinned by a continued attenuated response to WSSV challenge, which suggests that crabs can clear WSSV virions and prevent their replication. As this occurs alongside upregulation of immune and apoptosis-related transcripts in WSSV-recalcitrant crabs, such as caspase-1, hemocyanin, and cell death-inducing p53-target protein 1, this further indicates that the induction of programmed cell-death during early infection may be key to preventing WSD. The former has previously been shown to be upregulated in WSSV-injected *P. japonicus* (74).

In contrast, WSSV-replicating crabs exhibited many similarities with *P. vannamei* responses to WSSV challenge including large increases in transcriptional response which may correspond to expression changes associated with increased WSSV transcription and shifts in host transcription. The enrichment of GO terms associated with virus entry *via* clathrin mediated endocytosis and the cytoskeleton suggest that in these crabs WSSV is able to overcome the previously described mechanisms conferring increased WSSV resistance. Additionally, downregulation of V-type ATPases previously reported in WSSV-injected *P. vannamei* and hypothesized to play a role in virus uncoating was observed (8, 75) This process may be regulated by the novel miRNA Cma-pmiR-14. Interestingly, the *C. maenas* transcriptional dataset also revealed upregulation of nuclear pore complex transcripts and their posttranscriptional regulator Cma-pmiR-novel_20 which may act together to regulate nuclear invasion by WSSV. However, despite the transcriptional similarities of these species replicating WSSV, the absence of WSD-related mortalities in *C. maenas* suggests additional molecular or environmental factors may contributing to WSD resistance.

### 4.4 Responses to WSSV in susceptible versus recalcitrant species - a key to uncovering treatment targets

Investigation of the overlapping GO terms and KEGG pathways at each infection time point in *C. maenas* and *P. vannamei* revealed important differences in the response of these species to WSSV challenge. This is evidenced by the absence of overlapping enriched GO terms during early infection. Subsequently, in the late infection stages for each species, enriched processes in WSSV-replicating crabs were also distinct from shrimp, indicating that the mode of WSSV infection may differ between these two crustacean species. Novel enriched processes in the crab included ion transport, cell communication, nuclear pore complex and phosphotransferase activity which may contribute to manipulation of the host environment to make conditions less favorable for WSSV replication. The contrasting representation of several KEGG pathways represents possible key differences in the ability of these species to resist WSD. For example, the up-regulation of phosphatidylinositol signaling (dme04070) in WSSV-replicating *C. maenas* suggests an increased ability to sense pathogens, alert neighboring cells, initiate immune responses and regulate key processes involved in infection such as endocytosis. The up-regulation of oxidative phosphorylation (dme00190) was indicative of increased oxidative stress in susceptible species compared to resistant, and differences in ribosome (dme03010) representation illustrate the reduced hijack of crab machinery for increased translation and the production of virions. The similar down-regulation of the ribosome KEGG pathway in these species suggest a generic response to limit protein translation, which has differing efficacy and disease outcomes in each species. Overlap was observed between shrimp and crabs in the amino sugar and nucleotide sugar metabolism pathway (dme00520), however in shrimp this was up-regulated, possibly to accommodate for energy and biosynthetic requirements for WSSV replication and in crabs this pathway was down-regulated, highlighting a difference in how the metabolism of both organisms responds to the infection.

A larger number of miRNAs were differentially expressed in response to WSSV injection in *C. maenas* throughout the time course following WSSV injection. These results indicate that the increased expression of many miRNAs may occur as a result of WSSV challenge, due to their involvement in antiviral responses (Du et al., 2017; Wang and Zhu, 2017). However, due to the limited number of upregulated miRNAs in *C. maenas*, unless displaying evidence of WSSV replication, it could be hypothesized that these miRNAs enhance WSSV infection rather than limit it. This is supported by the detection of many significantly upregulated miRNAs within WSSV-replicating crabs compared to WSSV-recalcitrant crabs. Nevertheless, the further characterization of three overlapping differentially expressed miRNAs with opposing directions of change in shrimp and crabs (miR-279d, miR-133, and Pva-pmiR-118 and Cma-pmiR-14) in response to WSSV challenge should be explored.

### 4.5 Limitations

The findings presented herein support the hypothesis that WSD-susceptible and WSD-recalcitrant crustaceans exhibit distinct responses to WSSV challenge. However, the differences observed in these species must be considered within the content of the experimental designs utilized for each species. Firstly, whilst the use of wild-caught crabs offers the benefit of greater genetic heterogeneity than specific pathogen free lines [which were utilized for *P. vannamei* experiments (8)], we cannot exclude that these crabs may be responding to underling infections before or during WSSV exposure that may have impacted the transcriptional patterns measured. Additionally, the increased variation among crabs may also impact the ability to detect WSSV-specific transcription alterations, however, time matched control samples were analyzed for each time point to allow for the more precise detection of true positives. Secondly, control crabs were treated with saline solution and therefore it is not possible to separate transcriptional responses occurring as a result of metabolites within the WSSV inoculum rather than the virus itself. However, as limited transcriptional responses were observed during early time points within the crabs, this effect seems to be minimal.

Functional analysis studies rely on reliable annotations for genes and miRNAs and for many species, including crustaceans, high-quality annotated genome sequences are not available. In such cases, the annotation of novel *de novo* assembled transcripts relies on similarity of these genes and miRNAs to those published for closely-related species and the quality of the corresponding annotations. Critically, we assume function is conserved across species regardless of evolutionary distance but there is some uncertainty associated with this assumption. This is also the case for shrimp-crab cross species comparisons discussed within this study. Given this limitation, the number of false positive annotations were minimized by using a complete non-redundant protein database to query sequences and implementing stringent statistical thresholds (lower e-value) for annotation. miRNA interaction predictions also typically result in long lists with many false positives. To this end, only interactions predicted by more than one software tool, using conservative parameters such as a low minimum free energy score and alignment in the seed region were reported (76). Given these limitations we cannot rule out the presence of either false positives or false negatives, despite the mitigations made. Therefore, until functional studies are performed to validate our findings, the proposed interactions should be considered hypotheses, and representative of only a snapshot in time during the WSSV infection progression. However, collectively, these results demonstrate that the response of *C. maenas* does not follow the expected pattern for typical WSSV infection in susceptible species (8) providing important information for the understanding of resistance to WSSV in crustaceans.

### 4.6 Conclusions

This study addresses a significant gap in our understanding of the transcriptional (mRNA and miRNA) responses of a WSD-recalcitrant crustacean, *C. maenas*, to WSSV challenge. Our findings demonstrate that *C. maenas* elicits distinct responses compared to susceptible *P. vannamei* over time. These included early disruption to WSSV entry and movement by the downregulation of endocytosis transcript dynamin-1 and cytoskeletal transcripts. This key difference may subsequently result in a greater time window and opportunity for resistant species to alter the transcription of multiple miRNAs to fine-tune immune responses such as apoptosis, and to directly prevent translation of WSSV immediate early genes. Our study demonstrates the merit of comparative studies of crustaceans with differing WSD susceptibility in the identification of novel processes involved in the mechanisms of WSD recalcitrance. Further experimental validation of the transcripts and miRNA identified for their role in WSD resistance would provide a stepping stone for research into WSD prevention and treatments.

## Data availability statement

The datasets presented in this study can be found in online repositories. The names of the repository/repositories and accession number(s) can be found below: https://www.ncbi.nlm.nih.gov/genbank/, SRR14278211 - SRR14278323 and https://doi.org/10.6084/m9.figshare.21225128, as well as https://doi.org/10.6084/m9.figshare.21435831.

## Author contributions

RM, LB, BV, GS, CT, RA, and ES contributed to conception and all authors contributed to the experimental design of the study. LB and KB set up the crab WSSV exposure experiment. LB, AL, and AF extracted RNA and prepared sequencing libraries. AF and KM performed the sequencing. RM conducted the bioinformatics analysis, data curation, and visualization. CT, RA, and ES supervised the laboratory experiments and data analysis. RM wrote the manuscript, with subsequent input from all other authors. All authors contributed to the article and approved the submitted version.
