# Supplementary material for: Resistance to white spot syndrome virus in the European shore crab is associated with suppressed virion trafficking and heightened immune responses
Source: Front Immunol. 2022 Dec 27;13:1057421. doi: 10.3389/fimmu.2022.1057421 (PMC9831657; doi:10.3389/fimmu.2022.1057421)
Supplement: Supplementary file 12 [file DataSheet_1.pdf]

Supplementary Material

Resistance to White Spot Syndrome Virus (WSSV) in the European shore crab is associated with suppressed virion trafficking and heightened immune responses

Rebecca S. Millard[1,3*]&, Lisa K. Bickley[2,3]&, Kelly S. Bateman[1,3], Bas Verbruggen[2], Audrey Farbos[4], Anke Lange[2], Karen A. Moore[4], Grant D. Stentiford[1,3], Charles R. Tyler[2,3]†, Ronny van Aerle[1,3]†, Eduarda M. Santos[2,3*]†

[1] International Centre of Excellence for Aquatic Animal Health, Cefas Laboratory, Weymouth, United Kingdom

[2] Biosciences, College of Life and Environmental Sciences, University of Exeter, Exeter, United Kingdom

[3] Sustainable Aquaculture Futures, University of Exeter, Exeter, United Kingdom

[4] University of Exeter Sequencing Facility, Biosciences, College of Life and Environmental Sciences, University of Exeter, Exeter, United Kingdom

*** Correspondence:**
Rebecca S. Millard
rebecca.millard@cefas.gov.uk
Eduarda M. Santos
e.santos@exeter.ac.uk

† These authors contributed equally to this work and share senior authorship.

& These authors contributed equally to this work and share first authorship.

Contents

Supplementary Figure 1. Overview of mRNA-seq analysis bioinformatics workflow in WSSV-challenged *C. maenas.*

Supplementary Figure 2. Overview of miRNA-seq analysis bioinformatics workflow in WSSV-challenged *C. maenas.*

Supplementary Figure 3. miRNA length for control and WSSV-injected crabs.

Supplementary Figure 4. Bubble plot of significantly enriched GO terms (FDR q-val < 0.05) over time in *C. maenas* injected with WSSV.

Supplementary Data 1. Differentially expressed transcripts at 6 hours post injection between saline- and WSSV-injected *C. maenas*.

Supplementary Data 2. Differentially expressed transcripts at 12 hours post injection between saline- and WSSV-injected *C. maenas*.

Supplementary Data 3. Differentially expressed transcripts at 24 hours post injection between saline- and WSSV-injected *C. maenas*.

Supplementary Data 4. Differentially expressed transcripts at 48 hours post injection between saline- and WSSV-injected *C. maenas*.

Supplementary Data 5. Differentially expressed transcripts in late-stage WSSV-recalcitrant *C. maenas* compared to late-stage controls.

Supplementary Data 6. Differentially expressed transcripts in late-stage WSSV-replicating *C. maenas* compared to late-stage controls.

Supplementary Data 7. Reduced-redundancy *C. maenas* microRNAs predicted using miRDeep2 including mature, star and precursor sequences.

Supplementary Data 8. Significantly differentially expressed miRNAs in response to WSSV injection over time in *C. maenas*.

Supplementary Data 9. GO term enrichment among differentially expressed transcripts.

Supplementary Data 10. KEGG pathway representation in WSSV-injected *C. maenas*.

Supplementary Data 11. Table of possible interactions between differentially expressed *C. maenas* miRNAs and mRNAs presented in the current transcriptome.

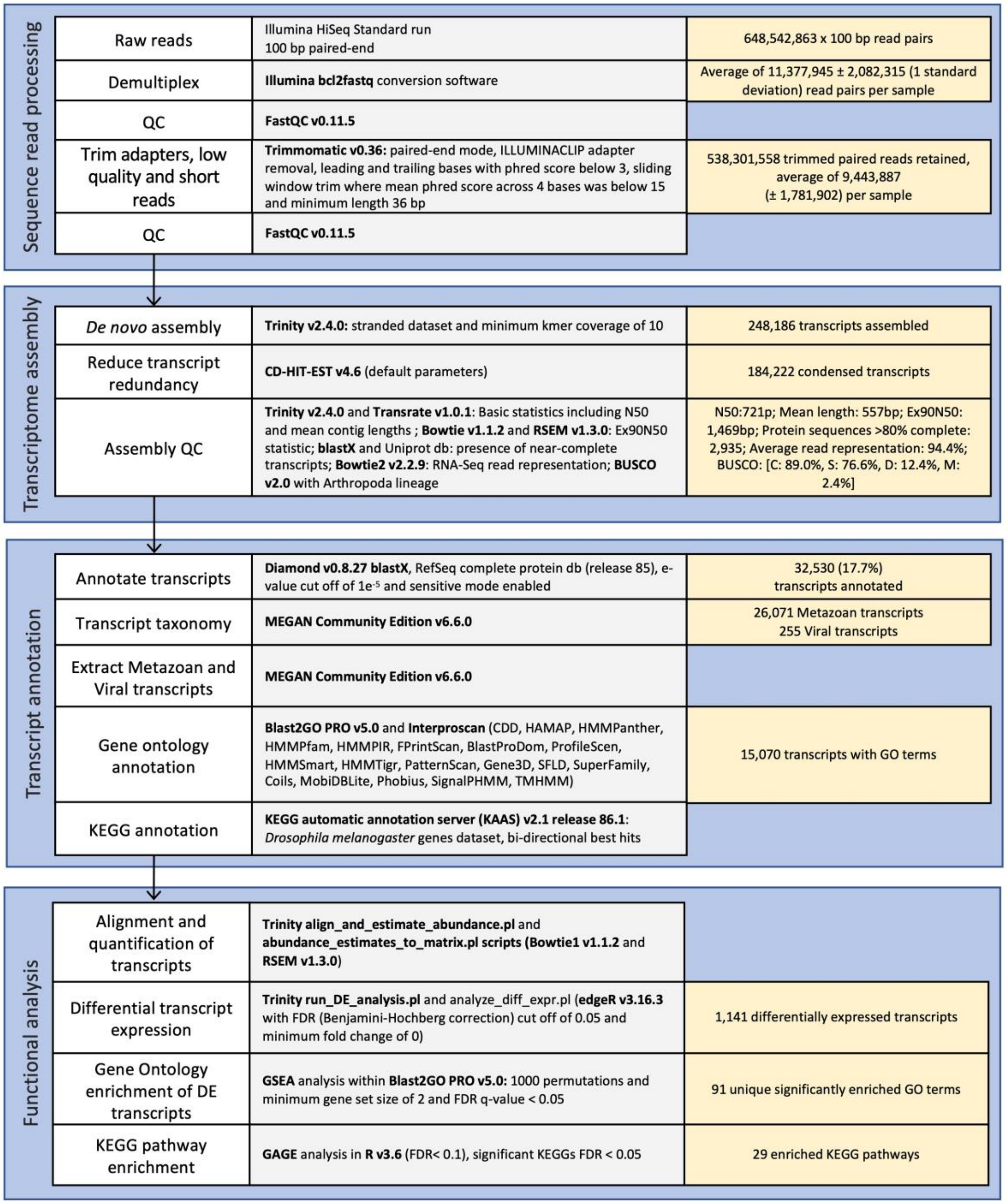

Supplementary Figure 1. Overview of mRNA-seq analysis bioinformatics workflow in WSSV-challenged *Carcinus maenas* (white boxes). Specific tools, scripts and parameters are depicted within grey boxes and a summary of the resulting outputs are in yellow boxes. The results depicted originate from downstream analysis of WSSV-injected crabs when grouped by their ability to replicate WSSV.

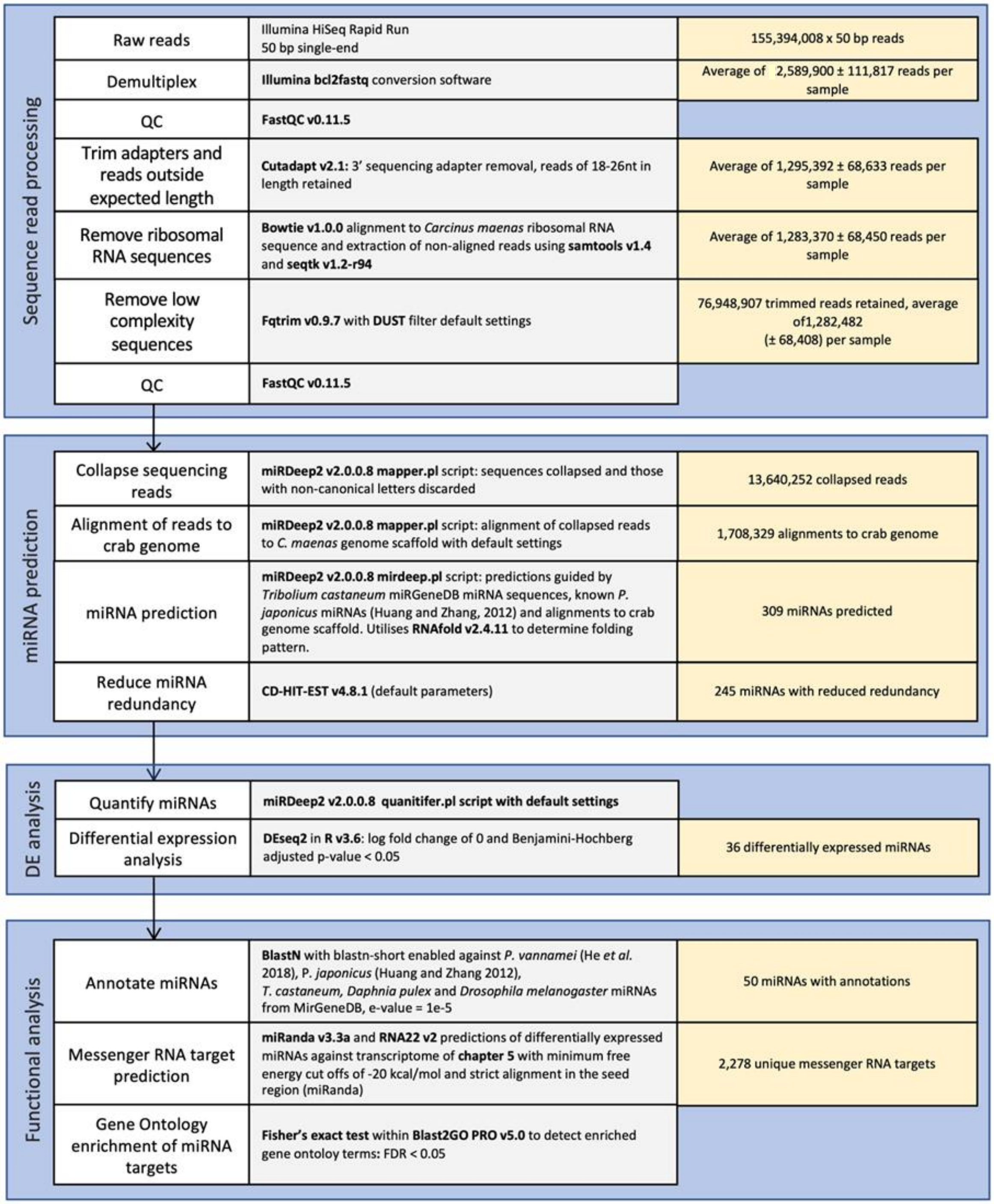

Supplementary Figure 2. Overview of miRNA-seq analysis bioinformatics workflow in WSSV-challenged *C. maenas* (white boxes). Specific tools, scripts and parameters are depicted within grey boxes and a summary of the resulting outputs are in yellow boxes. The results depicted originate from downstream analysis of WSSV-injected crabs when grouped by their ability to replicate WSSV.

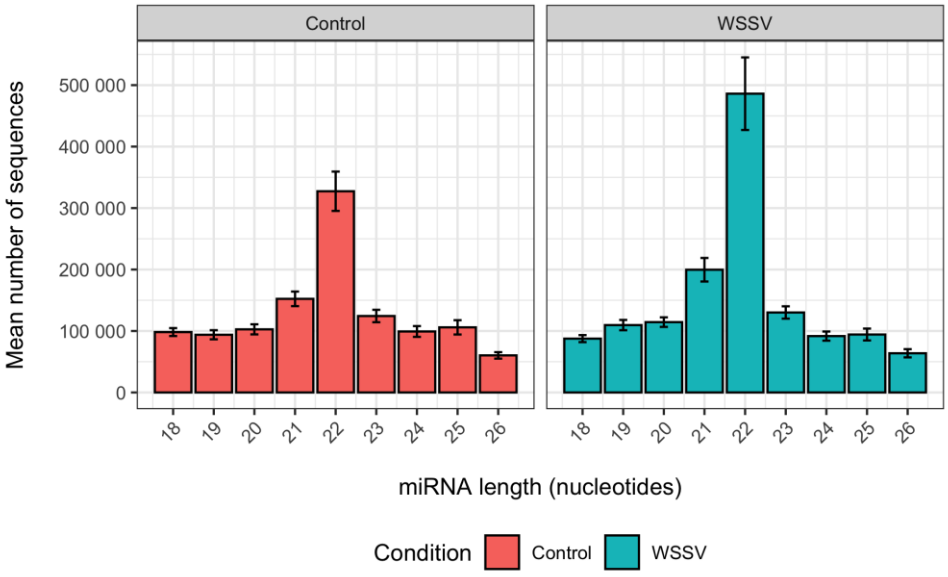

Supplementary Figure 3. miRNA length for (A) control and (B) WSSV-injected crabs. Average small RNA sequence length distribution for trimmed and filtered samples belonging to (a) saline-injected and (b) WSSV-injected *C. maenas* groups (error bars depict ± 1 standard error of the mean).

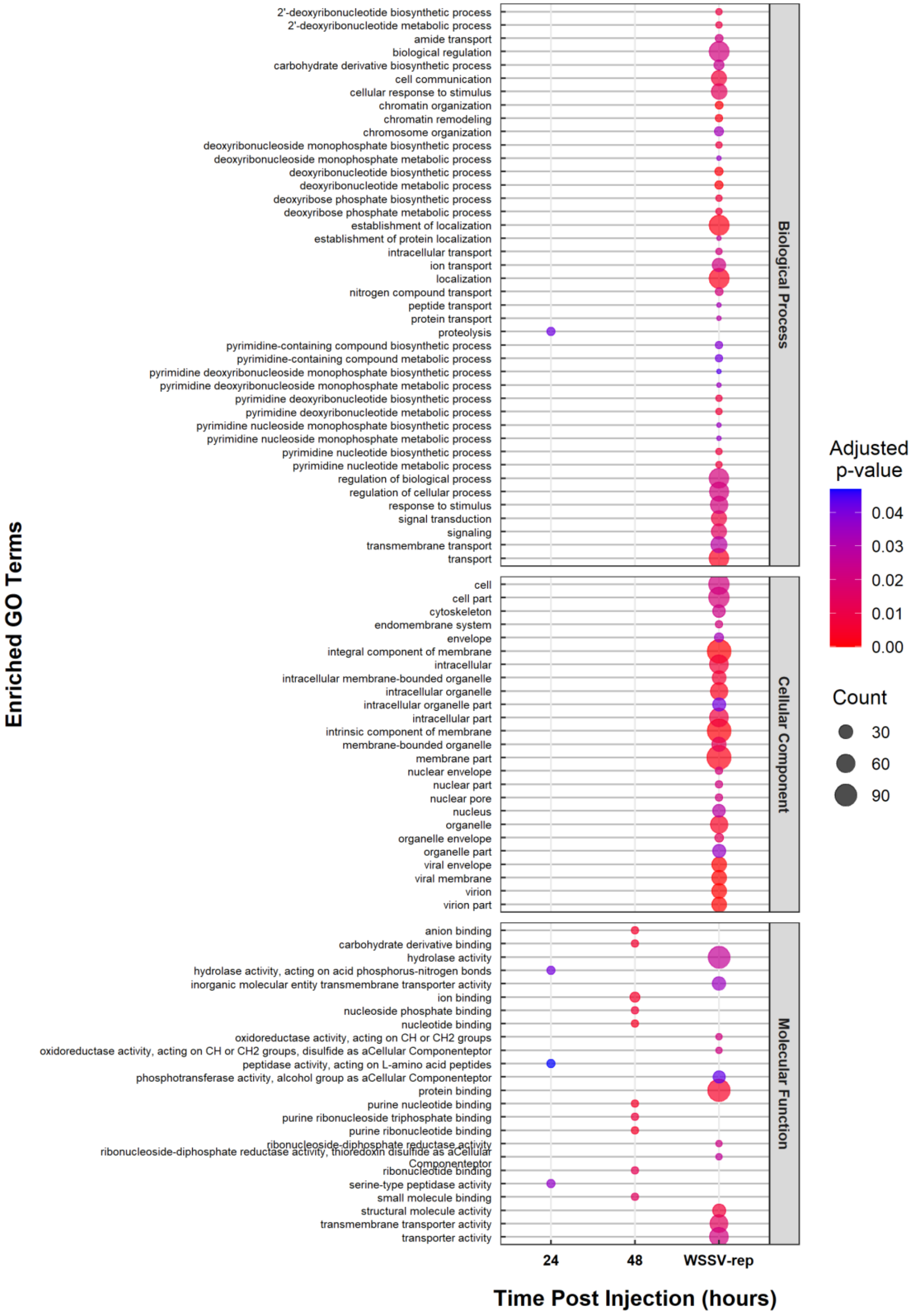

Supplementary Figure 4. Bubble plot of significantly enriched GO terms (FDR q-val < 0.05) over time in *C. maenas* injected with WSSV. GO terms are grouped by type depending on whether reflect

cellular components, biological processes or molecular functions. Point size represented the number of transcripts assigned to each GO term. The colour of each point reflects the significance of the GO term's enrichment (adjusted p value). In crabs, few processes are significantly enriched in direct time point comparisons, indicating that the virus is not subverting crab responses significantly to enhance infection. In crabs displaying evidence of WSSV replication, a greater number of processed were significantly enriched.
